# A kaleidoscope of photosynthetic antenna proteins and their emerging roles

**DOI:** 10.1093/plphys/kiac175

**Published:** 2022-04-21

**Authors:** Rameez Arshad, Francesco Saccon, Pushan Bag, Avratanu Biswas, Claudio Calvaruso, Ahmad Farhan Bhatti, Steffen Grebe, Vincenzo Mascoli, Moontaha Mahbub, Fernando Muzzopappa, Alexandros Polyzois, Christo Schiphorst, Mirella Sorrentino, Simona Streckaité, Herbert van Amerongen, Eva-Mari Aro, Roberto Bassi, Egbert J Boekema, Roberta Croce, Jan Dekker, Rienk van Grondelle, Stefan Jansson, Diana Kirilovsky, Roman Kouřil, Sylvie Michel, Conrad W Mullineaux, Klára Panzarová, Bruno Robert, Alexander V Ruban, Ivo van Stokkum, Emilie Wientjes, Claudia Büchel

**Affiliations:** Department of Biophysics, Centre of the Region Haná for Biotechnological and Agricultural Research, Faculty of Science, Palacký University, Olomouc 783 71, Czech Republic; Electron Microscopy Group, Groningen Biomolecular Sciences and Biotechnology Institute, University of Groningen, Groningen 9747 AG, The Netherlands; School of Biological and Behavioural Sciences, Queen Mary University of London, London, UK; Department of Plant Physiology, Umeå Plant Science Centre, Umeå University, Umeå 901 87, Sweden; Department of Physics and Astronomy and LaserLaB, Faculty of Science, Vrije Universiteit Amsterdam, Amsterdam 1081 HV, The Netherlands; Institute for Molecular Biosciences, Goethe University of Frankfurt, Frankfurt 60438, Germany; Laboratory of Biophysics, Wageningen University, Wageningen, the Netherlands; Department of Life Technologies, MolecularPlant Biology, University of Turku, Turku FI–20520, Finland; Department of Physics and Astronomy and LaserLaB, Faculty of Science, Vrije Universiteit Amsterdam, Amsterdam 1081 HV, The Netherlands; School of Biological and Behavioural Sciences, Queen Mary University of London, London, UK; Department of Botany, Jagannath University, Dhaka 1100, Bangladesh; Institute for Integrative Biology of the Cell (I2BC), CEA, CNRS, Université Paris-Sud, Université Paris-Saclay, Gif sur Yvette 1198, France; Université de Paris, Faculté de Pharmacie de Paris, CiTCoM UMR 8038 CNRS, Paris 75006, France; Dipartimento di Biotecnologie, Università di Verona, Verona, Italy; Photon Systems Instruments, spol. s.r.o., Drásov, Czech Republic; Department of Agricultural Sciences, University of Naples Federico II, Naples 80138, Italy; Institute for Integrative Biology of the Cell (I2BC), CEA, CNRS, Université Paris-Sud, Université Paris-Saclay, Gif sur Yvette 1198, France; Laboratory of Biophysics, Wageningen University, Wageningen, the Netherlands; Department of Life Technologies, MolecularPlant Biology, University of Turku, Turku FI–20520, Finland; Dipartimento di Biotecnologie, Università di Verona, Verona, Italy; Electron Microscopy Group, Groningen Biomolecular Sciences and Biotechnology Institute, University of Groningen, Groningen 9747 AG, The Netherlands; Department of Physics and Astronomy and LaserLaB, Faculty of Science, Vrije Universiteit Amsterdam, Amsterdam 1081 HV, The Netherlands; Department of Physics and Astronomy and LaserLaB, Faculty of Science, Vrije Universiteit Amsterdam, Amsterdam 1081 HV, The Netherlands; Department of Physics and Astronomy and LaserLaB, Faculty of Science, Vrije Universiteit Amsterdam, Amsterdam 1081 HV, The Netherlands; Department of Plant Physiology, Umeå Plant Science Centre, Umeå University, Umeå 901 87, Sweden; Institute for Integrative Biology of the Cell (I2BC), CEA, CNRS, Université Paris-Sud, Université Paris-Saclay, Gif sur Yvette 1198, France; Department of Biophysics, Centre of the Region Haná for Biotechnological and Agricultural Research, Faculty of Science, Palacký University, Olomouc 783 71, Czech Republic; Université de Paris, Faculté de Pharmacie de Paris, CiTCoM UMR 8038 CNRS, Paris 75006, France; School of Biological and Behavioural Sciences, Queen Mary University of London, London, UK; Photon Systems Instruments, spol. s.r.o., Drásov, Czech Republic; Institute for Integrative Biology of the Cell (I2BC), CEA, CNRS, Université Paris-Sud, Université Paris-Saclay, Gif sur Yvette 1198, France; School of Biological and Behavioural Sciences, Queen Mary University of London, London, UK; Department of Physics and Astronomy and LaserLaB, Faculty of Science, Vrije Universiteit Amsterdam, Amsterdam 1081 HV, The Netherlands; Laboratory of Biophysics, Wageningen University, Wageningen, the Netherlands; Institute for Molecular Biosciences, Goethe University of Frankfurt, Frankfurt 60438, Germany

## Abstract

Photosynthetic light-harvesting antennae are pigment-binding proteins that perform one of the most fundamental tasks on Earth, capturing light and transferring energy that enables life in our biosphere. Adaptation to different light environments led to the evolution of an astonishing diversity of light-harvesting systems. At the same time, several strategies have been developed to optimize the light energy input into photosynthetic membranes in response to fluctuating conditions. The basic feature of these prompt responses is the dynamic nature of antenna complexes, whose function readily adapts to the light available. High-resolution microscopy and spectroscopic studies on membrane dynamics demonstrate the crosstalk between antennae and other thylakoid membrane components. With the increased understanding of light-harvesting mechanisms and their regulation, efforts are focusing on the development of sustainable processes for effective conversion of sunlight into functional bio-products. The major challenge in this approach lies in the application of fundamental discoveries in light-harvesting systems for the improvement of plant or algal photosynthesis. Here, we underline some of the latest fundamental discoveries on the molecular mechanisms and regulation of light harvesting that can potentially be exploited for the optimization of photosynthesis.

## Teaser

The latest fundamental knowledge obtained on the light-harvesting mechanisms of the antenna proteins can be bridged to biotechnical optimization of photosynthesis.

## Introduction

Oxygenic photosynthesis is one of the most important biosynthetic pathways on Earth, sustained solely by solar energy. It is responsible for all oxygen production and for a global net primary production of about 1.05 × 10^17^ grams of fixed carbon per annum (Field et al., 1998). The primary processes involve the harvesting of solar energy, its conversion into chemical energy, and protection of the organism against photodamage. These processes lead to the synthesis of ATP and NADPH, which are used for assimilation of CO_2_. The primary reactions take place in the thylakoid membranes in cyanobacterial cells and the chloroplasts of eukaryotic photosynthetic organisms like plants and algae. The photosystems (PSs), responsible for photochemistry, are well conserved between the different organisms, whereas many different light-harvesting systems have evolved (Büchel, 2015; Croce et al., 2018). Membrane-extrinsic antennae like the phycobilisomes (PBSs) of cyanobacteria and red algae and the membrane-intrinsic proteins of the light-harvesting complex (Lhc) family in plants serve the same purpose: to efficiently harvest solar energy and transfer it to the PS reaction centers (RCs), where charge separation occurs in the specialized chlorophylls (Chls), fueling photosynthetic electron transport. To this end, the antenna proteins are arranged together with the PS cores in the so-called supercomplexes, enabling a high efficiency of transfer, but also its regulation.

The differences in antenna systems are accompanied by diversity in thylakoid membrane structure, ranging from almost homogeneous, parallel membranes, as in cyanobacteria, to the strong segregation into grana and stroma lamellae visible in vascular plants. Green algae also possess grana, although their grana stacks usually contain fewer membranes. In both green algae and plants, grana and stroma differentiation is accompanied by a segregation of the PSs (Anderson, 1986; Wietrzynski et al., 2020). PSII in a functional state is found almost exclusively in the grana and PSI in the stroma lamellae. Diatoms, as an example from the huge group of stramenopile algae, have thylakoids associated into bands of six membranes each. In this case, only enrichment of PSI in the outer membranes and of PSII in the inner four membranes of such a band could be demonstrated (Flori et al., 2017), but not such complete segregation as in vascular plants and green algae.

In order to optimize the use of the absorbed solar energy and at the same time to prevent damage to the organism under changing light conditions, all processes have to be tightly regulated. On the level of the primary reactions, the competition between light harvesting and photoprotection is of major importance: while under low-light conditions, plants and algae need to optimize energy harvesting, high-light conditions demand the harmless dissipation of excess energy. In vivo, changes in the incident light regime occur rather fast and frequently, enhancing the need for fast regulation. Eventually, the competition between light harvesting and photoprotection limits the yield of assimilation and by that the yield of products for human use.

Plants have been used by mankind mainly for nutrition, but also for medical purposes, and breeding has led to plant lines of considerable agronomical value. Lately, plants as well as algae have also come into use for biofuel production (Ruiz et al., 2016). For all purposes, high biomass or product yields are mandatory. Since the interplay between light harvesting and photoprotection is one of the limiting factors (Slattery et al., 2018), a thorough understanding of the regulation of light reactions is crucial to improve the organism’s ability to produce biomass for future biotechnological approaches and sustainable agriculture. This review thus first introduces the different photosynthetic systems of the diverse groups of organisms, including cyanobacteria, plants, green algae, and diatoms. It then deals with recent advances in understanding excitation energy transfer, photoprotection, and the plasticity of the thylakoid membrane.

## Natural diversity of PS supercomplexes

### PSII

**Figure 1:**
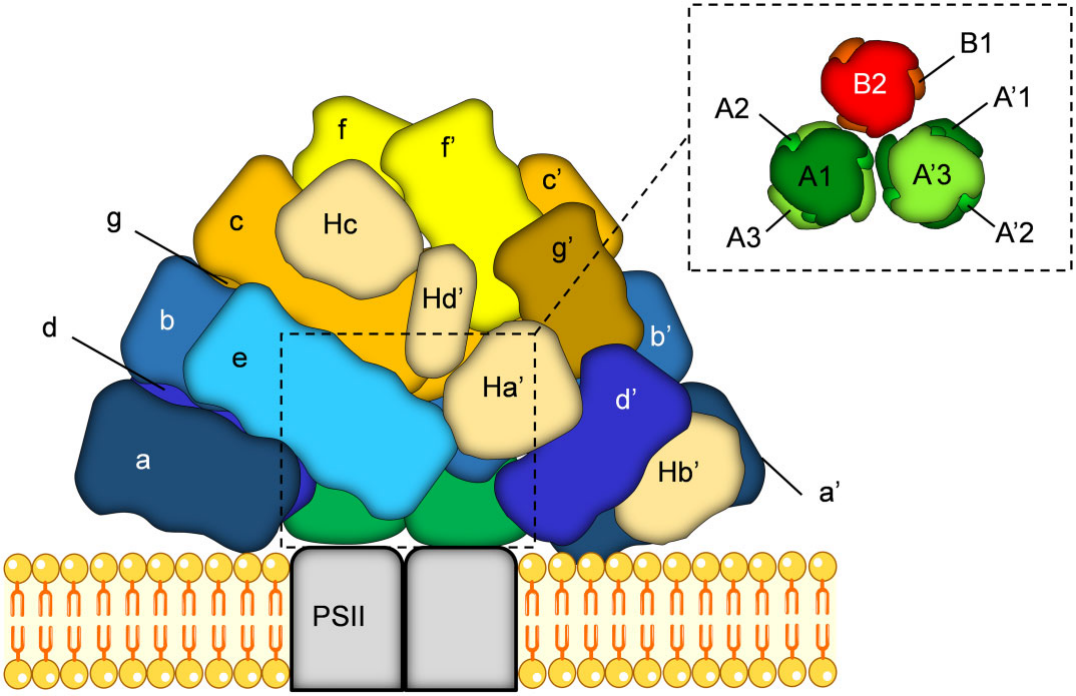
Organization of hemiellipsoidal PBS assemblies in the red alga *P. purpureum* (PDB entry: 6KGX). This is, at present, the highest resolution structure of a PBS deposited. Lowercase characters (a–g, a′–g′) mark the 14 peripheral rods, formed variably of phycoerythrin and phycocyanin complexes. Additional phycoerythrin hexamers are resolved, namely Ha′, Hb′, Hc, and Hd′ (their counterparts are not visible in this view). Minor individual phycoerythrin monomers and β subunits are omitted for clarity. The inset shows the structure of the PBS core, formed of one top cylinder (B) composed of 2 APC trimers (B1 and B2) and two basal cylinders. Each basal cylinder contains three APC trimers, namely, discs A1–3 and A′1–3. PSII, Photosystem II.

PSs are multi-subunit assemblies of light-harvesting antenna complexes and RC proteins. While the structure of reaction centers is conserved in photosynthetic organisms (Umena et al., 2011; Su et al., 2017; Pi et al., 2019; Sheng et al., 2019), the structure of antennae, the identity of bound pigments and, consequently their function in light-harvesting processes vary drastically. In cyanobacteria and red algae, the PBSs anchored to the cytoplasmic side of the thylakoid membranes serve as the major Lhc. PBSs are composed of water-soluble assemblies of open-chain tetrapyrrole chromophore-bearing phycobiliproteins and linker proteins that efficiently absorb light in a broad spectral range between 550 and 670 nm. Commonly, PBSs are hemidiscoidal or hemiellipsoidal structures, which consist of two key structural components: (1) a core substructure, which can be bicylindrical as in *Synechococcus* sp., but which is more typically tricylindrical as in *Synechocystis* sp. and (2) peripheral rods, protruding out from the core (Glauser et al., 1992). The rods can be composed of various pigmented proteins: C-phycocyanin (λ_max_ = 620 nm), phycoerythrin (λ_max_ = 565 nm), or phycoerythrocyanin (λ_max_ = 575 nm). The cores are instead built of a variable number of allophycocyanin (APC, λ_max_ = 650–670 nm) units (Figure 1). There are, however, large variations in the PBS organization and composition and deviations from this fundamental design depending on the species and growth conditions (Adir, 2005). The pigment–protein complexes of PBSs are organized as an energetic funnel, transmitting the absorbed energy via the rods to the core and ultimately to the RC) of the PSs with a very high quantum efficiency (Scott et al., 2006; Adir et al., 2020). Great progress has been made in recent years to solve the structure of PBSs at high resolution and reveal their docking site to PSs as well as possible regulatory sites (Zhang et al., 2017; Ma et al., 2020; Liu et al., 2021; Figure 1). Both cryo-electron microscopy (EM) structures from the Sui group, obtained from the red algae *Porphyridium purpureum* and *Griffithsia pacifica*, revealed the huge size of the PBSs and a convoluted rod architecture. In *P. purpureum*, >1,500 chromophores are bound, of which the majority are phycoerythrobilins (Ma et al., 2020).

Compartmental modeling analysis of picosecond time-resolved fluorescence studies revealed that, in intact cyanobacterial cells, the excitation energy from PBS can be rapidly distributed to the PSs (Tian et al., 2011; Acuña et al., 2018). The varying architecture and the composition of PBSs among cyanobacterial species seem to hardly affect the energy funneling from the PBSs to the PSs (Tian et al., 2011; Acuña et al., 2018; Akhtar et al., 2020; Biswas et al., 2020).

**Figure 2:**
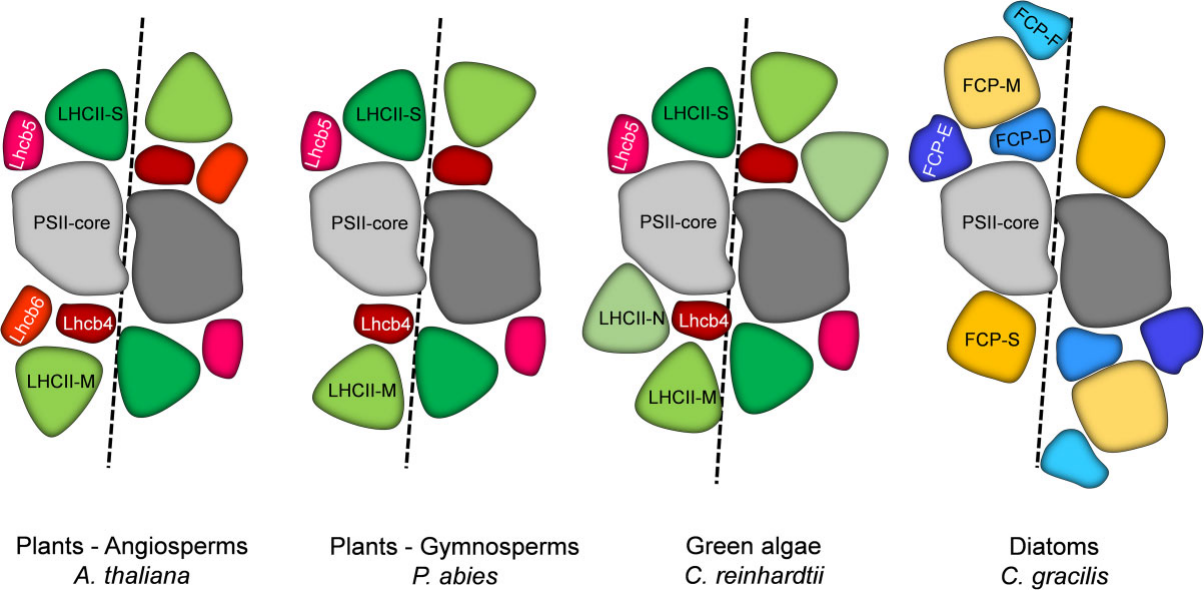
Organization of PSII supercomplexes in vascular plants, green algae and diatoms. The scheme is based on current structural data available (PDB entries: 5MDX, *A. thaliana*; 6KAF, *C. reinhardtii*; 6JLU, *C. gracilis*. The putative structure of *P. abies* PSII is shown as well [Kouřil et al., 2016]). PSII, photosystem II; Lhc, light harvesting complex; FCP, fucoxanthin chlorophyll protein.

**Figure 3:**
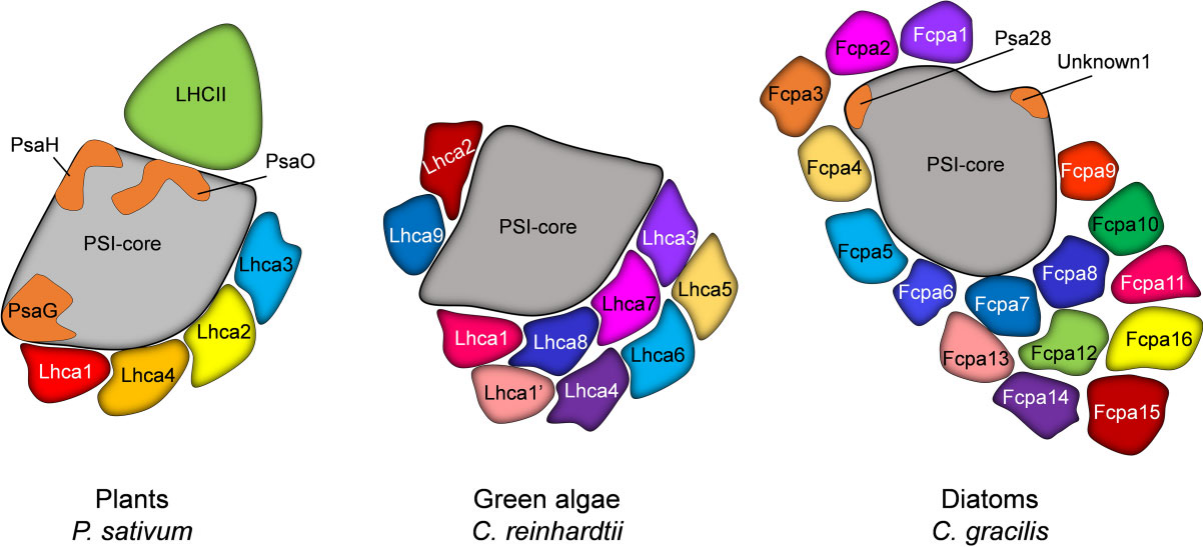
Organization of the PSI antenna system in vascular plants, green algae, and diatoms. The scheme is based on current structural data available (PDB entries: 5L8R, *Pisum sativum*; 6JO6, *C. reinhardtii*; 6L4U, *C. gracilis*). PSI, photosystem I; Lhc, light harvesting complex; Fcp, fucoxanthin chlorophyll protein; Psa, subunit of PSI.

In contrast to the bulky structure of PBSs in cyanobacteria, the Lhcs of plants and green algae binding Chl*a*, Chl*b*, and carotenoids are compact transmembrane proteins almost completely embedded in the thylakoid membrane (Figures 2 and 3). They are encoded by the multigenic Lhc family and can either serve the PSII (Lhcb) or the PSI cores (Lhca; Pan et al., 2020). In vascular plants, PSII binds the monomeric antenna proteins Lhcb4 (CP29), Lhcb5 (CP26), and Lhcb6 (CP24) and the trimeric LHCII complexes (encoded by combinations of Lhcb1-3; Jansson et al., 1997; Jansson, 1999). However, Lhcb6 is not present in green algae and has been lost during evolution in some subgroups of gymnosperms (Kouřil et al., 2016; Figure 2). The monomeric antenna proteins are essential for the proper energy transfer from LHCII to the PSII core (Caffarri et al., 2011; Dall’Osto et al., 2014). Especially, Lhcb4 (CP29) has been recently shown to play a pivotal role as an energy “channel” by harboring two low energy Chl*a* sites, one on the side of CP47 (Chlorophll Protein 47, a PSII core antenna, together with CP43) and one on the side of LHCII (Dall’Osto et al., 2020; Mascoli et al., 2020b). The trimeric antenna complexes of PSII can associate with the core complex differently; S trimers bind strongly, M trimers bind moderately and L trimers are loosely bound to PSII (Dekker and Boekema, 2005; Kouřil et al., 2018). S trimers are formed by different ratios of Lhcb1 and Lhcb2 proteins, and binding with the PSII core complex is supported by Lhcb4 and Lhcb5 monomers. M trimers contain a single copy of Lhcb3 and two copies of Lhcb1/2, and associate with the PSII core via Lhcb6 and Lhcb4 proteins (De Bianchi et al., 2008; Caffarri et al., 2004; Van Bezouwen et al., 2017; Su et al., 2017). In vascular plants, a PSII C_2_S_2_M_2_ supercomplex is formed by a dimeric core complex which binds two copies of S and M trimers each (Su et al., 2017; Van Bezouwen et al., 2017). In green algae, which lack the monomeric antenna protein Lhcb6, an additional N (naked) trimer is bound directly to the core complex without the involvement of this monomeric antenna protein, forming a C_2_S_2_M_2_N_2_ supercomplex (Figure 2; Drop et al., 2014; Shen et al., 2019; Sheng et al., 2019).

Fucoxanthin Chl-binding proteins (FCPs) are the antenna complexes in diatoms. They belong to the same protein family as LHCII and share several conserved structural and functional traits with their plant relatives, although they bind different chromophores (Premvardhan et al., 2010; Röding et al., 2018; Croce and van Amerongen, 2020). Recently, the structure of an FCP from *Phaeodactylum tricornutum* was solved, showing a dimeric complex. FCP binds 7 Chl*a*, 2 Chl*c*, 7 fucoxanthin, and 1 diadinoxanthin per monomer, bringing the Car/Chl ratio close to one, much higher than in green algae or plants (Wang et al., 2021). Besides these dimers, also trimeric FCPs were found in *P. tricornutum* (Gundermann et al., 2013; Lepetit et al., 2017). In other species, *Cyclotella meneghiniana* and *Thalassiosira pseudonana*, trimeric FCP complexes prevailed (Röding et al., 2018; Arshad et al., 2021)*.* Here, FCP complexes of higher oligomeric state (Büchel, 2003), loosely bound to the PSs but highly abundant in the thylakoid membrane, are present as well (Calvaruso et al., 2020). The structure of the PSII-FCP supercomplexes revealed a unique organization of the Lhcs, with three monomeric FCPs in locations different from the minor antenna of plants (Nagao et al., 2019; Pi et al., 2019; Arshad et al., 2021), and, depending on the species, tetrameric (*Chaetoceros gracilis*) or trimeric FCPs (*T.* *pseudonana*).

### PS I

The core of the PSI reaction center (Figure 3) is composed of the subunits PsaA and PsaB that also act as inner antenna similar to CP43 and CP47 in PSII (Jordan et al., 2001; Cardona, 2017). A large contribution to light harvesting in PSI comes directly from the pigments bound to the core, 96 Chls *a* and 22 carotenoids (Jordan et al., 2001). Nevertheless, in plants, the core complex of PSI binds four additional monomeric antenna proteins referred to as LHCI (Lhca1-4), which form a belt on one side of the PSI core (Ben-Shem et al., 2003; Mazor et al., 2017). LHCI in plants bind in total of 45 Chls *a*, 12 Chls *b*, and 13 carotenoids, increasing the absorption cross-section of the PSI core by ∼60% (Qin et al., 2015). Lhca3 and Lhca4 bind Chls that absorb at wavelengths >700 nm, which is substantially lower in energy than most Chls of the core (Croce and van Amerongen, 2020). This implies an uphill energy transfer between these complexes and the core energy trap, which, for this reason, is slower than for Lhca1 and Lhca2. Despite its large size and the peculiar red Chls in LHCI, the PSI–LHCI complex is extremely fast and efficient, and performs photochemistry with a quantum efficiency of almost 98%, making PSI the most efficient photochemical energy converter (Nelson, 2009; Croce and van Amerongen, 2013; Le Quiniou et al., 2015).

PSI in the green alga *Chlamydomonas reinhardtii* is larger than that in plants and contains two additional Lhca subunits on the PsaH/PsaG side and four Lhca subunits bind as an additional moon-shaped arc on top of the inner belt found in plants (Qin et al., 2019). The additional antenna proteins bind in total over 65 extra Chls, increasing the absorption cross-section by 41% in comparison to that of plants, without affecting the excitation energy transfer and trapping time (Le Quiniou et al., 2015). This is possible because the Chl red forms in green algae have higher energy levels in comparison with those in plants, leading to a similar average overall trapping time in both organisms (Le Quiniou et al., 2015). Recently, it was shown that in a colony-forming green alga, *Botryococcus braunii*, PSI can bind Lhca subunits at all known binding positions as seen in green algae and vascular plants, which in turn maximizes the antenna size while maintaining a high energy transfer efficiency (van den Berg et al., 2020).

The PSI antenna of diatoms is even larger than that of green algae, although the amount of antenna complexes bound seems to be dependent on species and/or culture conditions (Figure 3). Equivalent to plant LHCI, the FCPI complexes connected to the PSI core are in monomeric form. FCPI are arranged in several layers around PSI, with up to 24 antenna subunits present (Nagao et al., 2020; Xu et al., 2020; Arshad et al., 2021). In the PSI structure reported in (Nagao et al., 2020), the number of Chls bound to the full FCPI complement is 128, making its light-harvesting cross-section effectively larger than the PSI core. In diatoms, a large antenna system and binding of unique pigments are necessary for efficient light harvesting and energy dissipation, which ultimately assures the success of diatoms in the aqueous environment.

## Regulation of energy transfer and trapping in the photosynthetic membrane

### Adaptations to excessive sunlight

The adaptability of photosynthetic organisms to diverse and changeable environmental conditions derives from intricate molecular mechanisms to regulate photosynthesis. Often, sunlight intensity exceeds the capacity of the photosynthetic machinery and the unused, potentially harmful excitation energy enhances the probability of the generation of reactive oxygen species. Nonphotochemical quenching (NPQ) ensures a fast control of the amount of light energy conveyed to reaction centers and catalyzes the dissipation of the energy absorbed in excess (Demmig-Adams et al., 2014). NPQ is a process of dynamic adaptation to light intensity, in most cases strongly reliant on feedback cues generated by an overly active photosynthetic machinery. The major component of NPQ in plants is indeed called energy-dependent quenching (qE), since it depends on the increase of thylakoid transmembrane ΔpH, driven by photosynthetic electron transport (Ruban, 2016). It is the quickest, promptly reversible component of NPQ and, in plants, it relies on the activation of a small transmembrane protein called PSII subunit S (PsbS), while being enhanced by the activity of the xanthophyll cycle enzymes, which reversibly convert violaxanthin to zeaxanthin during high-light exposure. Ultimately, these changes lead to the formation of dissipative interactions within the pigment network, shortening the excitation lifetime. This section aims at giving a brief overview of the topic and its most recent developments, but is not exhaustive. Readers are referred to the reviews of (Bennett et al., 2019; Ruban and Wilson, 2020; Bassi and Dall’Osto, 2021) for further information.

Many aspects of the molecular mechanism of NPQ remain obscure, due to the experimental challenges posed by the protein-congested, highly plastic thylakoid membrane. In particular, the site and the mechanism of qE in plants have been long-standing open questions.

A breakthrough in pinpointing the qE site was attained with the creation of Arabidopsis (*Arabidopsis thaliana*) mutants lacking either all minor antennae (“No minor” mutants [*NoMs*]; Dall’Osto et al., 2014, 2017) or major LHCII complexes (Nicol et al., 2019; Nicol and Croce, 2021). The analysis of qE in these mutants unequivocally revealed the importance of major LHCII in the process of energy dissipation: while the absence of minor antennae causes changes in the kinetics of the qE onset (Dall’Osto et al., 2017), the absence of LHCII causes a major decrease of the extent of reversible quenching that reaches only up to 40% of the wild-type value (Nicol et al., 2019). In *NoM*, the noticeable transient NPQ relaxation occurring shortly after illumination was attributed to the absence of minor antennae, since this phenotype was also observed in koCP29 mutants (De Bianchi et al., 2008; Dall’Osto et al., 2017), and wild-type NPQ kinetics were re-established upon complementation with the CP29 wild-type sequence (Guardini et al., 2020; Bassi and Dall’Osto, 2021). By complementing koCP29 with site-directed CP29 mutant sequences on Chl-binding sites, the quenching ability was assigned to specific domains in CP29 (Guardini et al., 2020). However, ΔpH and electron transport reactions are impaired in the absence of minor antennae (De Bianchi et al., 2008; Townsend et al., 2018). A spectroscopic examination of the *NoM* mutant suggested that a smaller ΔpH extent, and consequently an impaired low-pH-dependent accumulation of zeaxanthin, rather than the lack of specific quenching sites in minor antennae, are the causes of the transient NPQ relaxation in the *NoM* mutant (Townsend et al., 2018). Saccon et al. (2020c) treated the NoM mutant with the chloroplast protein synthesis inhibitor lincomycin, producing plants lacking all minor antennae and most of the reaction center proteins. Provided that zeaxanthin is present, qE in these plants was shown to reach the same amplitudes as in wild-type. This model offered insights into the zeaxanthin- and PsbS-mediated regulation of the LHCII function, highlighting the allosteric nature of these factors. Both components modulate qE sensitivity to ΔpH, shifting it to smaller values and thereby offering plants a dynamic control of qE based on metabolic and environmental cues. LHCII, PsbS, and ΔpH are clearly the definers of qE in plants (Pawlak et al., 2020; Saccon et al., 2020c; Nicol and Croce, 2021). How much the minor antennae also contribute to quenching is still being argued (Townsend et al., 2018; Guardini et al., 2020; Bassi and Dall’Osto, 2021). So far, a mutant entirely lacking Lhcb1, Lhcb2, and Lhcb3 polypeptides has not been reported (Andersson et al., 2003; Nicol et al., 2019), which would help to quantify their contribution during qE. Many aspects of the role of PsbS during qE remain to be understood, despite recent advances. In vitro and in silico works have shown that specific luminal residues of the protein are involved in sensing pH (Li et al., 2004; Liguori et al., 2019; Krishnan-Schmieden et al., 2021). Since PsbS does not stably bind pigments and is therefore unlikely to be a site of quenching itself, its action could be to transduce lumen acidification to the bulk of LHCII, either by direct binding (Wilk et al., 2013; Correa-Galvis et al., 2016; Sacharz et al., 2017), or by transiently modifying the lipid environment of LHCII (Daskalakis et al., 2019).

The photoprotective role of Lhcs relies on their ability to switch between a long-lived state functional for light-harvesting and a short-lived (quenched) state that dissipates the absorbed energy as heat (Moya et al., 2001). The molecular mechanism of quenching and the nature of the photoprotective switch, however, remain hard to disentangle, due to the intrinsic nonfluorescent and short-lived character of the quencher, as well as the occurrence of experimental artifacts (Van Oort et al., 2018). This challenge was faced by investigating the quenching mechanism in monomeric detergent-solubilized CP29 (Mascoli et al., 2019). Taking advantage of a substantial subpopulation of strongly quenched complexes and applying a target kinetic model of the transient absorption (TA) data, the spectroscopic signature associated with the quenching mechanism was extracted and assigned to excitation energy transfer from Chls to a dark state of lutein 1, similarly to earlier results obtained on aggregated LHCII trimers (Ruban et al., 2007).

These findings open up two further questions: is the quenching mechanism identified in solubilized LHC also functional in the thylakoid membrane? And if so, how are the light-harvesting and quenched states regulated by changes of the membrane environment? En route to answering these questions, a recent study used TA measurements to address the quenched conformation of LHCII trimers immobilized in polyacrylamide gels to prevent clustering (Saccon et al., 2020a, 2020b). Similar to what was found in CP29 monomers, the signature of a carotenoid excited state, linked to the quenching of Chl singlet excited states, was detected. Consistent with this, a study based on time-resolved fluorescence experiments and advanced kinetic modeling led to the conclusion that the same quenching mechanism observed in monomeric CP29 is also active in CP29 oligomers in vitro (Mascoli et al., 2020a). Both studies also highlighted the importance of the membrane/protein environment in altering the equilibrium between quenched and unquenched LHC conformations. In line with this, a 2D spectroscopy approach on LHCII embedded in nanodisks showed a specific fine-tuning of pigment interactions in the membrane environment (Son et al., 2020a).

Finally, the role of zeaxanthin as an energy quencher during NPQ is still unclear. Recent reports of TA performed on thylakoid membranes suggest zeaxanthin may be involved as an energy quencher and contribute to energy dissipation with different mechanisms (Park et al., 2017, 2018; Bennett et al., 2019). In particular, the signature of a zeaxanthin radical cation was related to the formation of NPQ in vivo and consequently, a dissipative mechanism involving electron transfer to zeaxanthin has been proposed, based on the observation that the amplitude of NPQ in the *NoM npq1* mutant, lacking zeaxanthin, is similar to *NoM npq4*, lacking PsbS (Holt et al., 2005; Ahn et al., 2008; Dall’Osto et al., 2017; Park et al., 2017). The importance of this mechanism during NPQ is still under debate since some reports have shown that quenching can occur to a large extent even in the absence of the carotenoid (Li et al., 2009; Johnson et al., 2012; Saccon et al., 2020a; Nicol and Croce, 2021). LHCII function in vitro is hardly affected when zeaxanthin is bound (Xu et al., 2015; Tutkus et al., 2019; Son et al., 2020b), which may suggest a more indirect role for zeaxanthin in the thylakoid membrane (Havaux, 1998; Ruban and Johnson, 2010).

NPQ strategies have been diversified during evolution of photosynthetic lineages (Goss and Lepetit, 2015). While cyanobacteria contain a different antenna system, the PBS, photoprotection is also achieved by switching the antenna from a light harvesting to a quenched state. However, the underlying mechanism is remarkably different. The orange carotenoid protein (OCP) is a carotenoid-binding protein that can sense light intensity, thereby undergoing activation through a conformational change that occurs more often when blue illumination is increased in intensity (reviewed in Kerfeld et al., 2017 and Muzzopappa and Kirilovsky, 2020). In the activated form, the OCP binds to the PBS core, between the APC trimers (Harris et al., 2016) inducing the energy dissipation at the level of APC660 (Rakhimberdieva et al., 2010; Tian et al., 2011). However, the exact mechanism underlying this quenching and the role of the carotenoid of the OCP are still open questions. Recent works have focused on the diversity of OCP proteins. OCPs can be clustered in three subfamilies with different regulation features. For example, the canonical OCP1 is deactivated through interaction with another protein, named Fluorescence Recovery Protein (reviewed in Slonimskiy et al., 2020), while this additional regulation is not required for the OCP2 and OCPX (Bao et al., 2017; Muzzopappa et al., 2019). In addition, some cyanobacteria contain proteins coding for homologs of the OCP domains, the C-terminal domain homolog (CTDH), and the helical carotenoid protein (HCP; López-Igual et al., 2016; Melnicki et al., 2016). Although the in vivo role of these proteins is unknown, based on in vitro results it has been suggested that both could quench single oxygen, HCPs could also dissipate excess energy of PBS, and CTDH could be carotenoid carriers that ensure the proper carotenoid loading into HCPs (López-Igual et al., 2016; Muzzopappa et al., 2017; Harris et al., 2018).

Several proteins of the LHC superfamily acquired during evolution an exclusive role in photoprotective processes (Büchel, 2015; Giovagnetti and Ruban, 2018). Besides the already mentioned PsbS and OCP, present in vascular plants and cyanobacteria respectively, LHC stress-related (LHCSR) 1 and LHCSR3 protein complexes possess a prominent role during NPQ in the green algal lineage (Peers et al., 2009). Similar to PsbS, LHCSR proteins are able to sense luminal acidification and trigger the quenching response (Bonente et al., 2011; Liguori et al., 2013; Dinc et al., 2016; Tian et al., 2019). However, while PsbS is expressed constitutively in the thylakoids, LHCSRs expression is initiated upon illumination (Allorent et al., 2013; Polukhina et al., 2016). Moreover, while PsbS is unlikely to stably bind pigments (Dominici et al., 2002; Fan et al., 2015), several Chl and carotenoid molecules bind to LHCSRs, which have been proposed to be the site of NPQ (Bonente et al., 2011). Electron transfer between a carotenoid and a Chl within these complexes has been suggested as the quenching mechanism (Pinnola et al., 2016).

Another member of the LHCSR family, LHCX1, is critical for qE in diatoms, and quantitative variations in different ecotypes were suggested to influence the adaptability of diatoms to different environments (Bailleul et al., 2010; Buck et al., 2019). Structural data on LHCX1 are not available, and LHCX proteins were not resolved in the latest structure available for the PSII supercomplex (Nagao et al., 2019; Pi et al., 2019). Similar to LHCII in plants, the FCPs have been suggested to be the site where energy dissipation occurs, potentially leaving a secondary role to the LHCX proteins as transducers of the NPQ response (Elnour et al., 2018; Wang et al., 2021). Recent time-resolved fluorescence studies demonstrated that two quenching mechanisms are active in low-light acclimated diatoms, one in the proximity of the PSII core and one in the bulk FCP antenna (Chukhutsina et al., 2014; Taddei et al., 2018). Based on an analysis of an LHCX1 knockdown mutant, it was proposed that the core complex-associated NPQ is more effective in photoprotection. Acclimation to high light, on the contrary, enhances the antenna-related quenching component, facilitated by the accumulation of additional LHCX isoforms.

### Adaptations to different light spectra

A regulatory mechanism called state transitions functions to distribute the harvested light energy to the PSs. Overexcitation of PSI relative to PSII brings the organism to state I, in which the light harvested by PSII increases. On the other hand, overexcitation of PSII relative to PSI brings the organisms to state II, in which an increased amount of absorbed energy is directed toward PSI again (Allen, 2017; Johnson and Wientjes, 2019). In diatoms, no evidence of a similar mechanism has been found (Owens, 1986) and the physical process underlying state transitions appears to be different in land plants, green algae and cyanobacteria.

In plants and green algae, a similar mechanism regulates transition between states I and II based on the redox state of the plastoquinone pool (Allen et al., 1981). Reduction of the plastoquinone pool is sensed by the cytochrome *b6f* (Wollman and Lemaire, 1988; Vener et al., 1995), leading to the activation of the state transition kinase7 (Stn7). Upon its activation, Stn7 phosphorylates LHCII, which in turn dissociates from PSII and associates with PSI (state II) at the PsaK/H subunits, forming a PSI–LHCI–LHCII supercomplex (Bellafiore et al., 2005; Kouřil et al., 2005; Wientjes et al., 2013). In plants, the pool of LHCII involved in this transition is ∼20% (Allen, 1992; Vener 2007), and it seems as if it is LHCII heterotrimers containing two Lhcb1 and one Lhcb2 subunits are the important ones (Pietrzykowska et al., 2014) and that phosphorylation of Lhcb2 is more rapid (Leoni et al., 2013) than of Lhcb1. Recent results show that PSI antenna size can be largely extended by the association of multiple LHCII trimers. Benson et al. (2015) revealed that in addition to the “classical” binding at PsaK site, LHCII can associate with PSI via the Lhca antenna. In line with these results, Yadav et al. (2017) observed particles with more than one LHCII trimer attached with PSI at Lhca2/Lhca4, and Lhca2/Lhca3 sites. In contrast, in the green alga *C.* *reinhardtii*, a large portion of the LHCII is dissociated (Delosme et al., 1996) but only a minor fraction attaches to PSI, whereas the majority becomes quenched (Ünlü et al., 2014). Also here, as well as in the moss *Physcomitrium patens*, the Lhca antenna is involved in binding of LHCII trimers to PSI core complexes (Pinnola et al., 2018; Steinbeck et al., 2018). In *A.* *thaliana*, if the plastoquinone pool is oxidized the Stn7-kinase is deactivated and thylakoid-associated phosphatase38 (also known as protein phosphatase 1 [PPH1]) will dephosphorylate LHCII, which will then move to PSII (state I) (Pribil et al., 2010; Shapiguzov et al., 2010). In *C.* *reinhardtii*, two partially redundant phosphatases, CrPPH1 and PSII core phosphatase (CrPBCP), are involved in the regulation of state transitions (Cariti et al., 2020). However, there is always some LHCII bound to PSI, even in state I conditions, indicating that LHCII functions as a highly efficient PSI-antenna (Bos et al., 2019; Chukhutsina et al., 2020).

In cyanobacteria, as in plants, state transitions are triggered by a change in the redox state of the plastoquinone pool, but the molecular players that sense these changes are unknown. While the cytochrome *b6f* complex had been proposed as the redox sensor, a recent work had shown that it is not involved in cyanobacterial state transition (Calzadi et al., 2019). In the cyanobacterial species, *S*ynechococcus *elongatus* and *Synechocystis* sp PCC 6803, there is no redistribution of PBS, nor is there any spillover from PSII to PSI (Bhatti et al., 2020; Ranjbar Choubeh et al., 2020). However, in order to balance the excitation pressure between PSII and PSI, in state II, the PSII-core and not the PBS is quenched (Bhatti et al., 2020; Ranjbar Choubeh et al., 2020). Furthermore, in *S. elongatus*, two (sub) populations of PSII, namely quenched and unquenched exist, in both states I and II. The equilibrium between quenched and unquenched PSII is changed upon state transitions (Bhatti et al., 2021). In state I, a decoupling of PBS from PSI was observed, thereby altering the absorption cross section of PSI in *Synechocystis* sp PCC 6803 (Chukhutsina et al., 2015).

## Plasticity of the light-harvesting membranes

Light-harvesting membranes are highly flexible and dynamic systems that can exhibit extraordinary plasticity, particularly under harsh and unfavorable environmental conditions. The study of thylakoid membrane plasticity is therefore important for understanding the adaptation of the membrane and PSs to ever-changing natural conditions. Changes in the membrane structure and protein composition are common features during NPQ, state transitions, and acclimation processes.

### Toward high-resolution microscopy techniques to study photosynthetic adaptations

**Figure 4:**
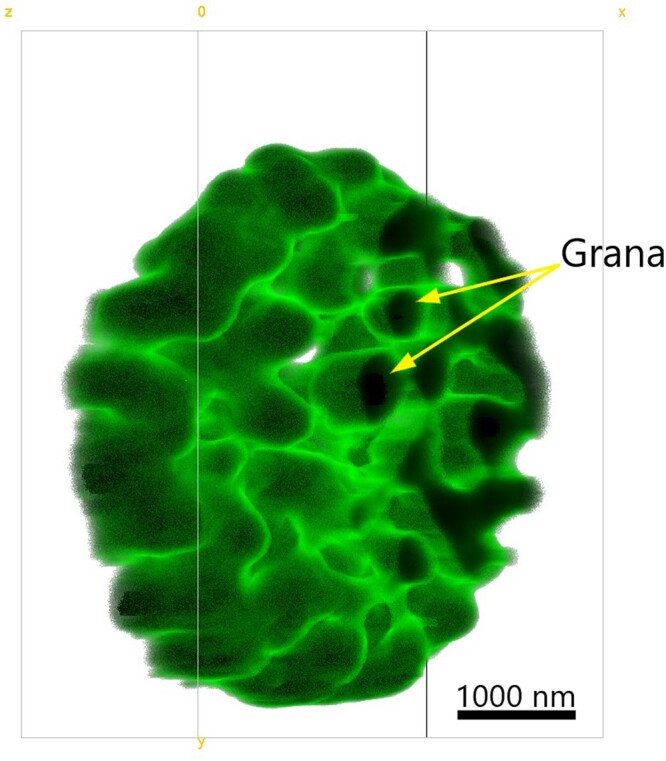
PRN image of the 3D *A. thaliana* thylakoid structure (turned by 45° anticlockwise around the *y-*axis with respect to data acquisition). Chloroplast was scanned by 70-nm *x* and *y* steps, and 300-nm *z* steps, collecting emission in 660–700 nm range. Sample was excited at 488 nm. 3D reconstruction was obtained from seven planes. Scale bar corresponds to 1,000 nm.

Current near-atomic resolution EM and high-resolution fluorescence imaging techniques complement each other for the studies of light-harvesting membrane plasticity. Photosynthetic membranes and their constituent subunits were investigated by EM for decades starting with purple bacteria (Tauschel and Drews, 1967). The evolution of EM techniques toward the atomic resolution of cryo-EM led to substantial discoveries of structural differences of photosynthetic complexes among organisms determined by their adaptation processes (Croce and van Amerongen, 2020). The potential of EM is further extended by cryo-electron tomography, which can provide structural information under the physiologically relevant conditions (Turk and Baumeister, 2020). Furthermore, cryo-electron tomography in combination with subvolume averaging has been used for visualization of PSs embedded in the isolated thylakoid membranes (Daum et al., 2010; Kouřil et al., 2011; Levitan et al., 2019; Arshad et al., 2021). The lack of visualization of dynamics within the sample, on the other hand, has accelerated the recent emergence of high-resolution fluorescence imaging techniques, which offer live-cell imaging possibilities. Considering the plant thylakoid membranes, structured-illumination microscopy and its derivatives have been recently shown to enable the observation of the nanometer-sized changes of the grana diameter and stacking induced by the adaptation of the organism to the varying light conditions (Wood et al., 2018, 2019). The 3D high-resolution imaging of the membrane structures is considered to bring even deeper insights into the dynamics of the photosynthetic apparatus. However, only a few attempts were reported up to now for 3D imaging of the membranes within the chloroplasts (Rumak et al., 2012). Recently, a promising high-resolution technique, pixel-reconstruction nanoscopy (PRN), was developed for live-cell imaging under nondamaging light intensities and minimal sample preparation. Figure 4 shows an example of PRN applications for the photosynthetic membranes—the 3D image of the thylakoid structure of an intact plant chloroplast. Green color in the image corresponds to the autofluorescence that originates mainly from the Chl molecules located in PSII (the darker the color, the higher the emission intensity) as emission from the PSI Chls is negligible at room temperature. This, by far the most accurate 3D image of native chloroplast, gives a hint that with current imaging tools it is possible to follow membrane adaptations in a whole plastid at physiological conditions at high precision. Moreover, in PRN, the ability to measure simultaneously various fluorescence emission channels uncovers an approach to study changes occurring at different sites of the membrane under altering environment of the organism. Additional techniques are emerging/consolidating that combine the use of microscopy with spectroscopy methods. One of these, fluorescence lifetime imaging, exploits the different Chl excited-state lifetime of PSII, PSI, and LHCs to visualize, among others, the distribution of PSs and the functional state of the antenna (e.g. during NPQ; Pascal et al., 2005; Iermak et al., 2016; Wientjes et al., 2017).

### Taking plasticity to the extreme: sustained NPQ in evergreens and the requirement for posttranslational modifications

The dynamics and flexibility of thylakoid membranes have been studied extensively in the membranes of angiosperms (Pribil et al., 2014; Lambrev and Akhtar, 2019). Recently, advances have been made in broadening the knowledge of photosynthetic complexes in gymnosperms. In particular, evergreen conifer species exhibit an extraordinary acclimation capacity to harsh boreal winters and are able to develop a so-called sustained form of protective NPQ (Öquist and Huner, 2003; Verhoeven, 2014). Several recent studies in members of Pinaceae, *Picea abies*, and *Pinus sylvestris* revealed a unique LHC composition (Kouřil et al., 2016; Grebe et al., 2019) and organization of PSII-LHCII supercomplexes and mega complexes (Kouřil et al., 2020). Although the physiological relevance of these complexes during winter acclimation is still under investigation, it has been suggested that specific posttranslational modifications, phosphorylation of Lhcb1 and PsbS proteins, are important prerequisites for the sustained NPQ (Grebe et al., 2020). Regarding the physiological relevance of grana destacking during winter and early spring, a recent study proposes its advantages in the molecular mechanism of the sustained NPQ process (Bag et al., 2020). Loss of appressed grana membranes increases the chances of close proximal contact between PSI and PSII, shown to result in direct energy transfer from PSII to PSI. Direct energy transfer from PSII to PSI is conferred to harmlessly dissipate the excess light energy in sub-zero temperatures and protect the photosynthetic machinery when linear electron flow is severely restricted, and the chances of photo-oxidative damage are high.

### The major sites of biogenesis of photosynthetic protein complexes

Besides the structural adaptability of photosynthetic protein complexes, their assembly and the subcellular location are part of the membrane’s plasticity. In chloroplasts, thylakoid biogenesis needs the import of the major photosynthetic proteins from the cytosol. Early work by cryo-electron tomography revealed thylakoid tip convergence zones in the green alga *C.* *reinhardtii* and the unicellular cyanobacterium *Synechocystis* sp. PCC6803, close to the envelope which might be the site of thylakoid biogenesis (Nickelsen and Zerges, 2013; Engel et al., 2015; Rast et al., 2019). Nothing is known so far from diatoms. Cyanobacteria have been considered good candidates to study the photosynthetic protein assembly sites, as they have less complicated thylakoid membrane arrangement compared to chloroplasts (Mullineaux and Sarcina, 2002; Mullineaux, 2014).

Cyanobacterial biogenic sites have been extensively explored by many research groups (Zak et al., 2001; Pisareva et al., 2011; Nickelsen and Rengstl, 2013; Weis et al., 2013; Selo et al., 2016; Rast et al., 2019). For the model cyanobacterium, *Synechocystis* sp. PCC 6803, the most commonly proposed sites of PS assembly are the thylakoid centers (Nickelsen and Zerges, 2013; Weis et al., 2013), that is, the regions where thylakoid membranes converge near the plasma membrane (Van De Meene et al., 2006). In a recent work, biogenic sites were investigated based on the localization of membrane-bound ribosomes in *Synechocystis* cells using cryo-electron tomography (Rast et al., 2019). Here, ribosomes were used as a marker of active protein translation. Only a small fraction of ribosomes was found near the thylakoid centers, while the majority was located at the thylakoid surfaces adjacent to the central cytoplasm. This suggests that the translation of photosynthetic protein subunits is not concentrated at the thylakoid centers. Furthermore, location of ribosomes can only provide a general idea regarding the overall protein synthesis. To specifically identify the start site of photosynthetic protein assembly, very recently a different approach was used, where mRNAs encoding the core subunits of PSI and II are probed in vivo in two model cyanobacteria (Mahbub et al., 2020). In this study, a single-molecule RNA Fluorescence in situ Hybridization (FISH) technique was used to visualize the target mRNAs in the cell. Results show that photosynthetic mRNAs mainly cluster as tight foci near the thylakoid surfaces adjacent to the central cytoplasm (Mahbub et al., 2020). In contrast, mRNAs encoding nonmembrane integral proteins locate further away from the thylakoid membrane. Treatments with protein translation inhibitors (puromycin and lincomycin) indicate that ribosome association with the mRNAs influences the distribution of mRNAs at the thylakoid surface. Therefore, these mRNA clusters adjacent to the cytosol-facing surface of the thylakoid membranes represent the major sites of translation of the core components of the PS (Mahbub et al., 2020).

The distribution pattern of PSII mRNA varies in different light conditions. Due to its extreme sensitivity to light, the D1 core protein of PSII undergoes a continuous damage and repair cycle (Nixon et al., 2010). In short-term high-light stress, D1 repair dominates over the de novo synthesis. In response to the stress, FISH signals distribute diffusely along the cytosol-facing surfaces of the thylakoid membranes, whereas in standard growth light conditions, the signal is more punctate (Mahbub et al., 2020). Therefore, mRNA-FISH probing can also detect the variable distribution of PSII synthesis, and thereby membrane plasticity in response to different environmental cues.

## Conclusions and future prospects

This review provides an update on the current understanding of photosynthetic light-harvesting and regulatory processes in vascular plants, algae, and cyanobacteria, whereby some questions still acquire more attention (see “Outstanding Questions”). The astonishing diversity in different evolutionary clades results in a kaleidoscope of solutions for light harvesting and photoprotection, in which the plasticity of the thylakoid membranes plays a crucial role. The recent development of accurate, high-resolution techniques to study the molecular processes during light harvesting, as well as the creation of informative mutants, has allowed fresh insights into this area.

For the most part, crop science has focused on agronomic approaches and breeding to improve plant architecture and light capture. However, annual increases in yields of the major crops in many parts of the world have plateaued, and new technological solutions must be explored (Blankenship et al., 2011; Ort et al., 2015). Improving photosynthesis through genetic engineering of light-harvesting processes is a possible solution to the development of new crop varieties with a higher yield potential (Kromdijk et al., 2016; Głowacka et al., 2018; Hubbart et al., 2018; Kirst et al., 2018; Simkin et al., 2019; Chen et al., 2020). Traits such as the protein PsbS and the carotenoid zeaxanthin have become prime examples of this. A strict control of energy dissipation within PSII in tobacco (*Nicotiana tabacum*) plants resulted in an increase of up to 15% in biomass in field conditions (Kromdijk et al., 2016), a finding that has more recently been translated to rice (*Oryza sativa*; Hubbart et al., 2018). PS antenna cross-section has also been revealed to be an important target for biotechnological improvement. A smaller size of the whole antenna complement has been often reported to positively affect biomass accumulation in plants and algae (Cazzaniga et al., 2014; Kirst et al., 2018). A precise understanding of antenna diversity and light-harvesting strategies is therefore pivotal for the identification of new targets for crop improvement.

Advances

- Major advances have been obtained in identifying the site and mechanism of NPQ, including disentangling the contribution of minor and major antennae in plants, identifying a Chl–carotenoid dissipative energy transfer pathway, and describing the mechanism of OCP-mediated quenching in cyanobacteria.
- The structural organization of PSs in plants and algae has been resolved, enabling detailed studies on light harvesting and photoprotection in the supercomplexes.
- The mechanism of extreme light and temperature tolerance in evergreen conifers has been elucidated.
- Data have been obtained regarding the organization of supercomplexes in the thylakoid membranes and their biogenesis.

Outstanding questions

- What is the precise role of PsbS and zeaxanthin in regulating energy fluxes within PSII during NPQ?
- Is energy dissipation in the PBS the result of energy transfer to the carotenoid of the OCP? What are the roles of the OCP homologs CTDH and HCP?
- Which FCP proteins are involved in NPQ in diatoms?
- Which additional traits besides zeaxanthin accumulation and PsbS can be used to increase crop yield, also in view of increasing temperatures and more frequent droughts?
- How does antenna composition and size impact the productivity of major crop species?

## Funding

The authors acknowledge funding from the European Union’s Horizon 2020 research and innovation program under the Marie Skłodowska-Curie grant agreement No 675006 (SE2B: Solar Energy to Biomass–Optimization of light energy conversion in plants and microalgae).

*Conflict of interest statement.* None declared.

## Notes

R.A., F.S., and C.B. conceived the structure and content of the manuscript. C.S., F.S., and S.S. prepared all figures. All authors contributed to the writing. C.B. agrees to serve as the author responsible for contact and ensures communication.

The author responsible for distribution of materials integral to the findings presented in this article in accordance with the policy described in the Instructions for Authors (https://academic.oup.com/plphys/pages/general-instructions) is Claudia Büchel (C.Buechel@bio.uni-frankfurt.de).
